# Continuous Maternal Hemodynamics Monitoring at Delivery Using a Novel, Noninvasive, Wireless, PPG-Based Sensor

**DOI:** 10.3390/jcm10010008

**Published:** 2020-12-22

**Authors:** Yuval Atzmon, Efrat Ben Ishay, Mordechai Hallak, Romi Littman, Arik Eisenkraft, Rinat Gabbay-Benziv

**Affiliations:** 1 Obstetrics and Gynecology Department, Hillel Yaffe Medical Center, Hadera 38100, Israel; atzmony@gmail.com (Y.A.); MottiH@hy.health.gov.il (M.H.); 2 The Rappaport Faculty of Medicine, Technion, Haifa 32000, Israel; 3 Biobeat Technologies Ltd., POB 12272, Petah Tikva 44425, Israel; efratsand@gmail.com (E.B.I.); romi@bio-beat.com (R.L.); aizenkra@gmail.com (A.E.); 4 Institute for Research in Military Medicine, Faculty of Medicine, The Hebrew University of Jerusalem, and The Israel Defense Force Medical Corps, Jerusalem 9112102, Israel

**Keywords:** remote patient monitoring, noninvasive monitoring, delivery, maternal hemodynamics

## Abstract

Objective: To evaluate continuous monitoring of maternal hemodynamics during labor and delivery utilizing an innovative, noninvasive, reflective photoplethysmography-based device. Study design: The Biobeat Monitoring Platform includes a wearable wristwatch monitor that automatically samples cardiac output (CO), blood pressure (BP), stroke volume (SV), systemic vascular resistance (SVR), heart rate (HR) every 5 s and uploads all data to a smartphone-based app and to a data cloud, enabling remote patient monitoring and analysis of data. Low-risk parturients at term, carrying singletons pregnancies, were recruited at early delivery prior to the active phase. Big data analysis of the collected data was performed using the Power BI analysis tool (Microsoft). Next, data were normalized to visual presentation using Excel Data Analysis and the regression tool. Average measurements were compared before and after rupture of membranes, epidural anesthesia, fetal delivery, and placental expulsion. Results: Eighty-one parturients entered analysis. Epidural anesthesia was associated with a slight elevation in CO (5.5 vs. 5.6, L/min, 10 min before and after EA, *p <* 0.05) attributed to a non-significant increase in both HR and SV. BP remained stable as of counter decrease in SVR (1361 vs. 1319 mmHg⋅min⋅mL^−1^, 10 min before and after EA, *p <* 0.05). Fetal delivery was associated with a peak in CO after which it rapidly declined (6.0 vs. 7.2 vs. 6.1 L/min, 30 min before vs. point of delivery vs. after delivery, *p <* 0.05). The mean BP remained stable throughout delivery with a slight increase at fetal delivery (92 vs. 95 vs. 92.1 mmHg, *p <* 0.05), reflecting the increase in CO and decrease in SVR (1284 vs. 1112 vs. 1280 mmHg⋅min⋅mL^−1^, *p <* 0.05)with delivery. Placental expulsion was associated with a second peak in CO and decrease in SVR. Conclusions: We presented a novel application of noninvasive hemodynamic maternal monitoring throughout labor and delivery for both research and clinical use.

## 1. Introduction

Pregnancy, delivery, and the puerperium are characterized by ongoing major changes in maternal hemodynamics to adapt to the growing physiological demands [1,2,3]. Changes start as early as the first trimester and reach their peak during labor and delivery to adapt to the associated anxiety, exertion, pain, uterine contractions, uterine involution, and bleeding [4,5]. Understanding these changes is of paramount importance for allowing good clinical care in healthy parturients and especially in more challenging cases such as women with heart disease, preeclampsia, or peripartum hemorrhage. Despite advanced technology and numerous research contributions, data on maternal hemodynamics during labor are still inconsistent and lack validation. While some studies report rises in cardiac output (CO) beginning at the first stage of labor [2], others suggest that CO increment is mainly related to contractions at more advanced delivery [6]. In addition, the exact timing of CO decrease after labor is still uncertain [7,8]. Epidural anesthesia (EA) is known to influence maternal hemodynamics [1]; however, whether other events, such as rupture of membranes or placental separation, have any effect or reflection on maternal hemodynamics is not fully elucidated. Few methods for hemodynamic monitoring during labor and delivery exist. In the past, hemodynamic monitoring depended mainly on invasive techniques of pulmonary artery catheterization using the Fick method, dye dilution, or thermodilution [9,10,11,12]. However, the complexity of technique as well as the high risk for adverse events such as arrhythmias, pneumothorax, infection, thrombosis, and even death outweighed any presumed benefits except in very ill patients [13,14,15]. Echocardiography and tissue Doppler imaging are noninvasive alternatives for hemodynamic monitoring. Lung ultrasound can also serve as a helpful tool for detecting fluid intolerance. Although safe, these tools present logistical and cost issues and are operator dependent [16,17,18]. Over the recent years, technology has improved, allowing monitoring using whole-body bioimpedance and thoracic bioimpedance-based devices [19,20]. Both techniques are safe and easy to use; however, they still require maternal wiring, which is uncomfortable during labor. In addition, data on their correlation with invasive techniques are conflicting [21,22]. In this study, we assessed maternal hemodynamics during labor and delivery, by using continuous monitoring of cardiac output (CO), heart rate (HR), stroke volume (SV), systemic vascular resistance (SVR), and systolic-, diastolic-, and mean arterial blood pressure (SBP, DBP, and MAP) measured by a novel wearable, wireless, noninvasive reflective photoplethysmography (PPG) remote patient monitoring device

## 2. Materials and Methods

This was a prospective, observational, longitudinal data analysis study of continuous maternal hemodynamic monitoring using a novel PPG-based wearable device. The study was conducted at a single university-affiliated medical center between 1April 2019 and 28February 2020. The study was approved by the Hillel Yaffe Medical Center Institutional Review Board (HYMC-18-0101, NCT03838965) and each participant signed an informed consent form at enrollment.

### 2.1. Study Population

Women were eligible to participate if they were healthy, above 18 years old, and carrying term (37–42 gestational weeks), singleton pregnancy. Exclusion criteria included any major illness (e.g., heart disease, chronic hypertension, pregestational diabetes) or preeclampsia. Deliveries ending with cesarean section or subjects that removed the device prior to delivery were also excluded.

Women were enrolled in the delivery room prior to the onset of the active stage of labor. Following consent, women were given the device to be worn or their wrist. Initial calibration data including maternal age (years), height (cm), weight (Kg), and current heart rate and blood pressure were uploaded to the personal application provided with the device.

The wristwatch remained attached for at least two hours after delivery and was removed by study personnel prior to transferring the women to the maternity ward.

### 2.2. The PPG-Based Remote Patient Monitoring Device

**Figure 1:**
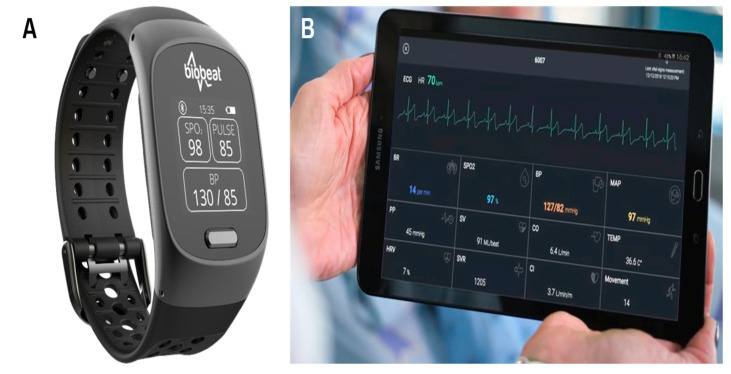
The Biobeat remote patient monitoring platform. (**A**)The wristwatch device. (**B**)Real-time measurement collected by the device.

The novel reflective PPG-based device (Biobeat Technologies Ltd., Petah Tikva, Israel) is a wearable, wireless, noninvasive medical-grade monitor that enables continuous remote patient monitoring, including the monitoring of maternal hemodynamics to assess cardiovascular changes during labor and delivery (Figure 1). Most commercially available PPG-based pulse oximeters transmit light in specific red and infrared wavelengths through the tissue. A detector measures the changing absorbance at each of the wavelengths, allowing it to determine the absorbance resulting from the pulsating arterial blood alone, excluding venous blood, skin, bone, muscle, and fat. Though PPG technology is commonly used for pulse oximetry, the device used in this study utilizes a unique reflective PPG technology in which the light source and sensor array are placed on the same side, and as the light is transmitted into the subject’s skin, part of it is reflected from the tissue to a photodiode detector. The PPG signal is collected with a high temporal and quantitative resolution, and by employing pulse wave transit time and pulse wave analysis techniques’ minute changes in tissue reflectance are captured, enabling measurement of numerous vital signs including HR, changes in blood pressure (BP), CO, SV, SVR, and more, every 5s. The PPG-based device has had both US Food and Drug Administration (FDA) clearance and a CE mark approval.

### 2.3. Data Collection

Maternal and neonatal data were prospectively collected upon admission and during labor and delivery. Data included maternal characteristics and medical background such as age, pre-gestational weight and height, weight gain during pregnancy, known cardiovascular or metabolic illness, medications, smoking, etc. Obstetric characteristics included parity, previous obstetric history, and current pregnancy follow-up (first and second trimester genetic screening, anatomy scan, glucose status, any hypertensive disorders). Timing of all interventions during labor was documented at the time of event including rupture of membranes (spontaneous or artificial), epidural anesthesia (EA), exact time of fetal delivery, and placental expulsion. Delivery outcomes included gestational age at delivery, neonatal birth weight, and immediate neonatal outcome. Maternal outcome such as peripartum hemorrhage or puerperal fever were documented until discharge. The measurement rate of the monitoring devices was once every five seconds, and the data were uploaded through a smartphone application to a secured data cloud environment, from which it was remotely analyzed. Vital signs included in the analysis were CO, HR, SVR, SV, SBP, DBP, and MAP.

### 2.4. Data Analysis

Big data analysis of the collected physiological data was performed using the Power BI analysis tool (Microsoft). Next, the data were normalized to visual presentation using Excel Data Analysis and the regression tool. The data were presented as continuous time-series trend lines, identifying behavioral changes in the vital signs. In all, the X-axes represented the monitoring timeline, while the Y-axes represented changes in the values along the timeline. Average measurements calculated from all obtained measurements within the time interval that was defined were compared before and after rupture of membranes (representing the first stage of labor), EA, fetal delivery (representing the second stage of labor), and placental expulsion.

### 2.5. Statistical Analysis

Data were presented visually and numerically. Average measurements in 10-min time intervals were compared before and after each of the events. Statistical analysis was performed using SPSS version 21.0 software (SPSS, Inc., Chicago, IL, USA). A paired samples *t*-test was used to assess the change in vitals prior to and following each of the events. *p <* 0.05 was considered significant. Intervention studies involving animals or humans, as well as other studies requiring ethical approval must list the authority that provided approval and the corresponding ethical approval code.

## 3. Results

Of the 106 parturients that were enrolled to the study, only 81 remained eligible for the study. Five parturients were excluded due to hypertensive disorders (4 preeclampsia and 1 with gestational hypertension), fourteen deliveries ended with an emergency cesarean section, and the other 6 parturients were excluded due to technical issues (removing the device prior to delivery or incomplete data transmission). Study cohort characteristics are presented in Table 1. The median maternal age was 30 (21–42) years and 24/81(29.6%) were nullipara. The median body mass index (BMI) was 29.2, range 20.7–48.1, Kg/m^2^. Continuous monitoring of all vitals was presented visually and numerically before and after EA, rupture of membranes, fetal delivery, and placental expulsion. Overall, 833,663 measurements were available for analysis. Video of ongoing continuous monitoring is available in the Supplementary File for selected women (Video S1–S5).

### 3.1. Epidural Anesthesia

**Figure 2:**
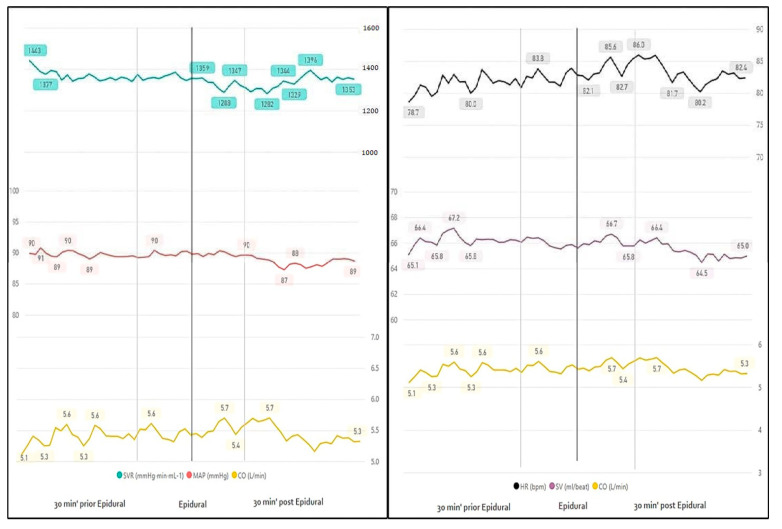
Continuous monitoring 30 min before and after epidural anesthesia. SVR—systemic vascular resistance; MAP—mean arterial blood pressure; *p* < 0.05 is marked in bold; CO—cardiac output.

Sixty-nine (85.2%) parturients underwent EA. Visually, we noted a slight elevation in CO (5.5 vs. 5.6, L/min, 10 min before and after EA, *p <* 0.05) followed by decreased values. CO elevation seems attributed to a non-significant increase in both HR and SV. Overall, blood pressure values remained stable due to counter decrease in SVR (1361 vs. 1319, mmHg⋅min⋅mL^−1^, 10 min before and after EA, *p <* 0.05) (Figure 2, Table 2).Continuous evaluation of measurements beyond 10 min after the epidural injection demonstrated a continuous decrease in SVR lasting only until 20 min after EA. The rest of the measurements were no longer statistically different (measured until 30 min after EA (Table 3).

#### 3.1.1. Rupture of Membranes

**Figure 3:**
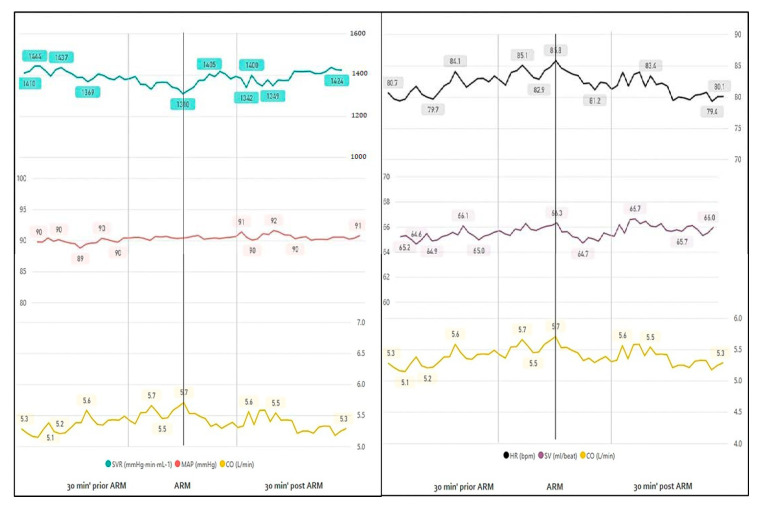
Continuous monitoring 30 min before and after rupture of membranes.

Over half of the parturients (50/81, 61.7%) underwent artificial rupture of membranes. For the entire cohort, the median time (m) from the rupture of membranes to delivery was 3 h and 42 min, range (r) 0:05–23:49 h (artificial rupture: *m* = 3:42, *r* = 0:15–23:49; spontaneous rupture: *m* = 3:20, *r* = 0:05–23:12). Analysis was based on measurements taken 10 min prior and after rupture of membranes. For all variables measured, the rupture of membranes had no impact over maternal hemodynamics (Figure 3, Table 2).

#### 3.1.2. Delivery

**Figure 4:**
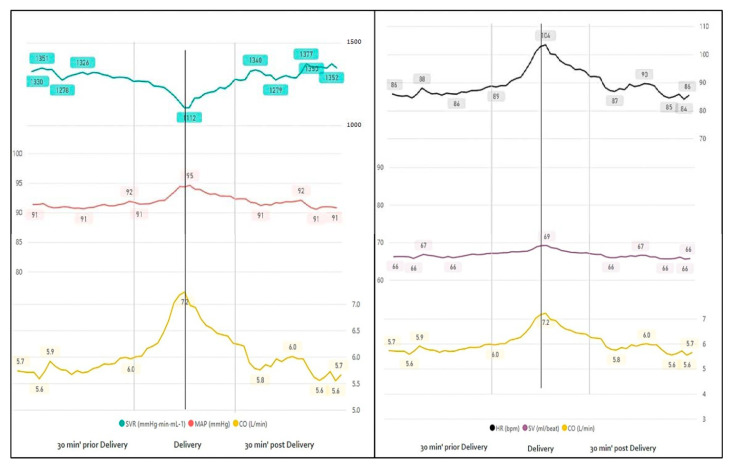
Continuous monitoring 30 min before and after delivery.

The greatest change in maternal hemodynamics appeared around fetal delivery. Cardiac output increased with labor, reaching a peak of 7.24 L/min at the point of fetal delivery, after which it rapidly declined (6.0 vs. 6.1 L/min, 30 min before and after delivery, *p <* 0.05) (Table 2, Figure 4). This was mainly due to marked changes in HR (88.8 vs. 104 vs. 90.5, bpm) and a slight change in SV (67 vs. 69 vs. 67.1, mL/beat), *p <* 0.05 for all. Mean BP remained clinically stable throughout delivery with a slight increase at fetal delivery (92 vs. 95 vs. 92.1, mmHg, *p <* 0.05), reflecting the increase in CO and decrease in SVR (1284 vs. 1112 vs. 1280, mmHg⋅min⋅mL^−1^, *p <* 0.05) with delivery (Figure 4, Table 2).

#### 3.1.3. Placental Expulsion

**Figure 5:**
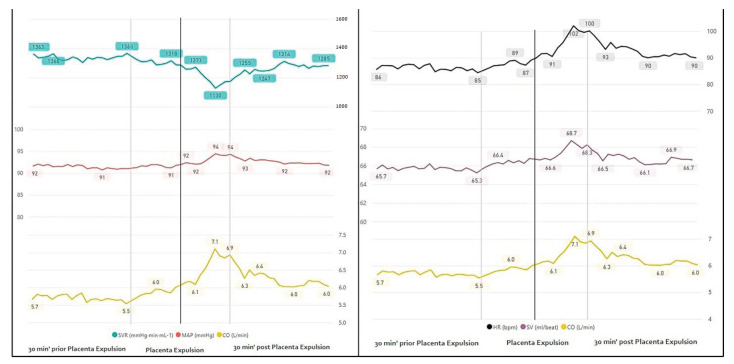
Continuous monitoring 30 min before and after placental expulsion.

Evaluation of maternal hemodynamics around placental expulsion revealed increased CO (5.9 vs. peak value at delivery 7.1 vs. 6.6, L/min, *p <* 0.05) most probably due to increased HR (88.5 vs. 96, bpm) without any difference in SV, 10 min before and after the event. Similarly, blood pressure values remained stable with a decrease in SVR (1294 vs. 1130 vs. 1200 mmHg⋅min⋅mL^−1^, *p <* 0.05), probably countering the CO increment. To note, the median time to placental expulsion was 8 min, consistent with peak values presented visually (a range of 0 to 57 min from delivery) (Figure 5, Table 2).

## 4. Discussion

In this observational longitudinal study, we presented continuous maternal hemodynamic monitoring of low risk parturients undergoing vaginal deliveries using a novel PPG-based remote patient monitoring device. Our study presented the longitudinal adjustments of HR, SV, CO, SVR, SBP, DBP, and MAP sampled every 5 s throughout labor and delivery as well as their adaptation to “events” during labor and delivery: EA, rupture of membranes, fetal, delivery and placental expulsion. According to our results: (1) EA causes only minor changes in SVR and CO with stable blood pressure values up to 30 min after EA; (2) no maternal hemodynamics changes were documented following rupture of membranes; (3) fetal delivery was the point of maximal changes for all measured variables with a rapid onset significant reversal of changes after fetal delivery; and (4) placental expulsion was associated with a second peak in CO and HR, and a decrease in SVR, thus maintaining stable blood pressure values.

Thus far, studies investigating maternal hemodynamics during labor and delivery have yielded conflicting results, especially regarding magnitude and timing of hemodynamic adaptations. Previous studies suggested inconsistent results on the effect of EA on maternal hemodynamics during labor [19,23,24,25,26]. EA induces a sympathetic blockade that causes vasodilatation and a decrease in venous return to the heart, which can result in maternal hypotension. In addition, abrupt onset of pain relief may trigger a reduction in blood pressure. However, these effects are seen in only some of the studies and reported clinically in only 0–14% of laboring women receiving EA [23,24,25,26,27]. Recently, Ashwal et al. [19] compared maternal hemodynamics following vaginal and cesarean deliveries determined by a whole body bioimpedance-based device. They found a significant decrease in cardiac index and in MAP measured before and after EA. However, their sampling was continuous for only a 6-min interval before and after EA without standardization regarding the time interval before and after onset of anesthesia. In addition, data on fluid overload given prior to EA and its effect on measurements were not documented. In our study, we have documented the longitudinal changes in maternal hemodynamics following EA in a 5-s interval and found that blood pressure remained stable until 30 min after EA. This serves not only as a proof of concept for an easy, noninvasive, and accurate technique for longitudinal assessment of maternal hemodynamics, but also has clinical significance in terms of abandoning the fluid overload given prior to EA to allow real time adaptation of fluid management and fetal monitoring. The simplicity of this technology will enable us to further broaden our knowledge of maternal hemodynamics and individualize the management of high-risk parturients and complicated deliveries.

Rupture of membranes, although potentially relieving the tension within the uterus, did not impact any of the variable measured.

Like previous reports [1,2,3,4,5,6], the peak of changes was noted with fetal delivery. CO increased from early labor to the second stage, reaching 7.2 L/min with fetal delivery. This is most probably related to the strong contractions causing autotransfusion of uterine blood into the maternal systemic circulation, thus increasing the preload. Thirty minutes after fetal delivery, there was already a marked decrease in CO. Unlike the robust changes in CO, MAP remained relatively unchanged with only a mild increment found in the second stage and fetal delivery. To note, changes in blood pressure may differ between studies, as it is dependent upon many variables usually uncontrolled for between parturients such as: Duration and intensity of uterine contractions, maternal position, subjective maternal pain and anxiety, and timing of maternal pushing.

Placental expulsion was associated with a second peak in CO and HR that appeared minutes after delivery. Despite the rise in CO, MAP remained relatively unchanged due to a compensating decrease in SVR.

Our study benefitted from its longitudinal methodology with continuous monitoring of hemodynamic data every 5 s during labor and delivery. This was possible using the novel PPG-based technology that was previously validated as well as adjusted to specific maternal anthropometrics. In addition, as participants were derived from a single center, a standard approach was applied for all deliveries.

Our study was limited due to the small sample size. In addition, as our study was observational, we could not control measurements for variables such as maternal positioning, fluid management, maternal anxiety, timing of bearing down, etc. Lastly, we did not evaluate the change in hemodynamic parameters in different settings of maternal and obstetrical complications such as previous cardiovascular disease and acute bleeding.

## 5. Conclusions

In this study we utilized a novel PPG-based device to evaluate maternal hemodynamic adjustment during labor and delivery. Further studies should focus on hemodynamic monitoring in parturients with preexisting cardiovascular or obstetrical complications such as preeclampsia and use these data to define normal and abnormal values for creation of safety protocols during labor and delivery.

## Figures and Tables

**Table 1:** Study cohort characteristics (*n* = 81).

Maternal age, years	30 (21–42)
Advanced maternal age (>35 years)	16 (19.7)
BMI, Kg/m^2^	29.2 (20.7–48.1)
Class II–III BMI (>35 Kg/m^2^)	36 (44.4)
Nulliparity	24 (29.6)
Gestational diabetes	14 (17.3)
Induction of labor	33 (40.7)
Epidural anesthesia	69 (85.2)
Artificial rupture of membranes	50 (61.7)
Gestational age at delivery in weeks	39.4 (37–41.9)
Birth weight in grams	3265 (2615–4494)

Data are presented as *n* (%) for categorical values and median (range), for continuous variables. BMI—body mass index.

**Table 2:** Trends in vital signs during labor and delivery.

**Epidural Anesthesia**			
	**10 min prior**	**10 min after**	***p*-Value**
HR	81.9	84.0	0.0727
SVR	1361	1319	0.0177
CO	5.5	5.6	0.0079
SV	66.6	66.9	0.0733
MAP	89.4	89.8	0.2464
SBP	123.5	123.9	0.4724
DBP	72.2	72.7	0.1822
**Rupture of MEMBRANES**			
	**10 min prior**	**10 min after**	***p*-Value**
HR	88	85	0.3502
SVR	1233	1282	0.2601
CO	5.8	5.5	0.3113
SV	65.9	64.4	0.1309
MAP	87.9	86.6	0.0614
SBP	120	119.7	0.0832
DBP	71.6	70	0.0526
**Delivery**			
	**30 min prior**	**30 min after**	***p*-Value**
HR	88.8	90.5	0.0002
SVR	1284	1280	0.0000
CO	6.0	6.1	0.0003
SV	67.0	67.1	0.0001
MAP	92.0	92.1	0.0059
SBP	125.5	126.1	0.0071
DBP	75.2	75.1	0.0061
**Placental Expulsion**			
	**10 min prior**	**10 min after**	***p*-Value**
HR	88.5	96	0.0040
SVR	1294	1200	0.0464
CO	5.9	6.6	0.0049
SV	66.8	67.7	0.2182
MAP	92.2	93.4	0.6398
SBP	126	127	0.9658
DBP	75	76	0.6557

Numbers represent mean values of all obtained measurements within the time interval presented; CO—cardiac output; HR—heart rate; SV—stroke volume; SVR—systemic vascular resistance; SBP—systolic blood pressure; DBP—diastolic blood pressure; MAP—mean arterial blood pressure.

**Table 3:** Trends in vital signs 20 and 30 min before and after epidural anesthesia.

Epidural Anesthesia			
	**10 min Prior**	**10–20 min after**	***p*-Value**
HR	81.9	83.5	0.1648
SVR	1361	1317.8	0.0240
CO	5.5	5.5	0.0854
SV	66.6	66.4	0.4080
MAP	89.4	88.3	0.5243
SBP	123.5	122.2	0.3459
DBP	72.2	71.3	0.7622
	**10 min Prior**	**20–30 min after**	***p*-Value**
HR	81.9	81.6	0.1913
SVR	1361	1348.2	0.4167
CO	5.5	5.3	0.1128
SV	66.6	65.7	0.8424
MAP	89.4	87.9	0.0929
SBP	123.5	121.7	0.2257
DBP	72.2	70.9	0.0709

Numbers represent meanvalues of all obtained measurements within the time interval presented; CO—cardiac output; HR—heart rate; SV—stroke volume; SVR—systemic vascular resistance; SBP—systolic blood pressure; DBP—diastolic blood pressure; MAP—mean arterial blood pressure.

## Supplementary Materials

The following are available online at https://www.mdpi.com/2077-0383/10/1/8/s1. Video S1: HR: In timeline and stage and events during labor and delivery; Video S2: SVR: In time line and stage and events during labor and delivery; Video S3: MAP: In time line and stage and events during labor and delivery. Video S4: CO: In timeline and stage and events during labor and delivery; Video S5: CO: In time line and stage and events during labor and delivery.

## References

[1] Ouzounian J.G., Elkayam U. (2012). Physiologic changes during normal pregnancy and delivery. Cardiol. Clin..

[2] Hunter S., Robson S.C. (1992). Adaptation of the maternal heart in pregnancy. Br. Heart J..

[3] Chapman A.B., Abraham W.T., Zamudio S., Coffin C., Merouani A., Young D., Johnson A., Osorio F., Goldberg C., Moore L.G. (1998). Temporal relationships between hormonal and hemodynamic changes in early human pregnancy. Kidney Int..

[4] Meah V.L., Cockcroft J.R., Backx K., Shave R., Stöhr E.J. (2016). Cardiac output and related haemodynamics during pregnancy: A series of meta-analyses. Heart.

[5] Duvekot J.J., Cheriex E.C., Pieters F.A., Menheere P.P., Peeters L.H. (1993). Early pregnancy changes in hemodynamics and volume homeostasis are consecutive adjustments triggered by a primary fall in systemic vascular tone. Am. J. Obstet. Gynecol..

[6] Maruta S. (1982). The observation of thematernal hemodynamics during labor and cesarean section. Nippon Sanka Fujinka Gakkai Zasshi.

[7] Mahendru A.A., Everett T.R., Wilkinson I.B., Lees C.C., McEniery C.M. (2014). A longitudinal study of maternal cardiovascular function from preconception to the postpartum period. J. Hypertens..

[8] Morris E.A., Hale S.A., Badger G.J., Magness R.R., Bernstein I.M. (2015). Pregnancy induces persistent changes in vascular compliance in primiparous women. Am. J. Obstet. Gynecol..

[9] Sorensen M.B., Bille-Brahe N.E., Engell H.C. (1976). Cardiac output measurement by thermal dilution: Reproducibility and comparison with the dye-dilution technique. Ann. Surg..

[10] Fegler G. (1957). The reliability of the thermodilution method for determination of the cardiac output and the blood flow in central veins. Q. J. Exp. Physiol. Cogn. Med. Sci..

[11] Driul L., Meroi F., Sala A., Delrio S., Pavoni D., Barbariol F., Londero A., Dogareschi T., Spasiano A., Vetrugno L. (2020). Vaginal delivery in a patient with severe aortic stenosis under epidural analgesia, a case report. Cardiovasc. Ultrasound.

[12] Swan H.J., Ganz W., Forrester J., Marcus H., Diamond G., Chonette D. (1970). Catheterization of the heart in man with use of a flow-directed balloontipped catheter. N. Engl. J. Med..

[13] Ventura H.O., Taler S.J., Strobeck J.E. (2005). Hypertension as a hemodynamic disease: The role of impedance cardiography in diagnostic, prognostic, and therapeutic decision making. Am. J. Hypertens..

[14] Harvey S., Harrison D.A., Singer M., Ashcroft J., Jones C.M., Elbourne D., Brampton W., Williams D., Young D., Rowan K. (2005). PAC-Man study collaboration. Assessment of the clinical effectiveness of pulmonary artery catheters in management of patients in intensive care (PAC-man): A randomized controlled trial. Lancet.

[15] Peters S.G., Afessa B., Decker P.A., Schroeder D.R., Offord K.P., Scott J.P. (2003). Increased risk associated with pulmonary artery catheterization in the medical intensive care unit. J. Crit. Care.

[16] Vetrugno L., Dogareschi T., Sassanelli R., Orso D., Seremet L., Mattuzzi L., Scapol S., Spasiano A., Cagnacci A., Bove T. (2020). Thoracic ultrasound evaluation and B-type natriuretic peptide value in elective cesarean section under spinal anesthesia. Ultrasound J..

[17] Arbeid E., Demi A., Brogi E., Gori E., Giusto T., Soldati G., Vetrugno L., Giunta F., Forfori F. (2017). Lung Ultrasound Pattern Is Normal during the Last Gestational Weeks: An Observational Pilot Study. Gynecol. Obstet. Investig..

[18] Easterling T.F., Benedetti T.J., Schmucker B.C., Millard S.P. (1990). Maternal hemodynamics in normal and preeclamptic pregnancies; a longitudinal study. Obstet. Gynecol..

[19] Ashwal E., Shinar S., Orbach-Zinger S., Lev S., Gat R., Kedar L., Pauzner Y., Aviram A., Yogev Y., Hiersch L. (2018). The Hemodynamics of Labor in Women Undergoing Vaginal and Cesarean Deliveries as Determined by Whole Body Bioimpedance. Am. J. Perinatol..

[20] Lavie A., Ram M., Lev S., Blecher Y., Amikam U., Shulman Y., Avnon T., Weiner E., Many A. (2018). Maternal cardiovascular hemodynamics in normotensive versus preeclamptic pregnancies: A prospective longitudinal study using a noninvasive cardiac system (NICaS™). BMC Pregnancy Childbirth.

[21] Young J.D., McQuillan P. (1993). Comparison of thoracic electrical bioimpedance and thermodilution for the measurement of cardiac index in patients with severe sepsis. Br. J. Anaesth..

[22] Gotshall R.W., Wood V.C., Miles D.S. (1989). Comparison of two impedance cardiographic techniques for measuring cardiac output in critically ill patients. Crit. Care Med..

[23] Nachman D., Gepner Y., Goldstein N., Kabakov E., Ishay A.B., Littman R., Azmon Y., Jaffe E., Eisenkraft A. (2020). Comparing Blood Pressure Measurements Between a Photoplethysmography-Based and a Standard Cuff-Based Manometry Device. Sci. Rep..

[24] Grant G.J., Susser L., Cascio M., Zakowski M.I. (1996). Hemodynamic effects of intrathecal fentanyl in nonlaboring term parturients. J. Clin. Anesth..

[25] Palmer C.M., Van Maren G., Nogami W.M., Alves D. (1999). Bupivacaine augments intrathecal fentanyl for labor analgesia. Anesthesiology.

[26] Simmons S.W., Taghizadeh N., Dennis A.T., Hughes D., Cyna A.M. (2012). Combined spinal-epidural versus epidural analgesia in labour. Cochrane DatabaseSyst. Rev..

[27] Grangier L., de Tejada B.M., Savoldelli G.L., Irion O., Haller G. (2020). Adverse side effects and route of administration of opioids in combined spinal-epidural analgesia for labour: A meta-analysis of randomised trials. Int. J. Obstet. Anesth..

