# Supplementary material for: Continuous Maternal Hemodynamics Monitoring at Delivery Using a Novel, Noninvasive, Wireless, PPG-Based Sensor
Source: J Clin Med. 2020 Dec 22;10(1):8. doi: 10.3390/jcm10010008 (PMC7793094; doi:10.3390/jcm10010008)
Supplement: Supplementary file 1 [file jcm-10-00008-s001.zip › jcm-1036363.docx]

**Supplementary Materials:**

A patient, 26 years old, G4P3 39 + 6 weeks, admitted to the delivery room following spontaneous onset of delivery and entered the study at 00:07 am. Her pre-gestational BMI was 28 Kg/m^2^. She received epidural anesthesia at 00:25, followed by artificial rupture of membranes at 03:35. She reached full dilatation at 04:35 and delivered a healthy 3270-g baby boy at 04:41 am. Placental expulsion occurred spontaneously at 04:45. Videos attached below:

Video S1: HR In time line and stage and events during labor and delivery.

Video S2: SVR In time line and stage and events during labor and delivery.

Video S3: MAP In time line and stage and events during labor and delivery.

Video S4: CO In time line and stage and events during labor and delivery.

Video S5: CO In time line and stage and events during labor and delivery.
